# Pandemic (H1N1) 2009 and Seasonal Influenza A (H3N2) in Children’s Hospital, Australia

**DOI:** 10.3201/eid1710.101670

**Published:** 2011-10

**Authors:** Gulam Khandaker, David Lester-Smith, Yvonne Zurynski, Elizabeth J. Elliott, Robert Booy

**Affiliations:** The University of Sydney, Sydney, New South Wales, Australia

**Keywords:** influenza, H1N1, H3N2, pandemic, children, viruses, seasonal viruses, Australia, letter

**To the Editor:** We read with interest the report by Carcione et al. of clinical features of pandemic influenza A (H1N1) 2009 and comparison of these with 2009 seasonal influenza infection in a population-based study from Western Australia (*1*). Here we share our experience of hospitalizations for influenza in a tertiary care children’s hospital in Sydney, New South Wales, Australia, during the 3 peak influenza seasons of the last decade.

During the 2009 Southern Hemisphere single influenza wave (June–September), we prospectively studied every child <15 years of age who was hospitalized with laboratory-confirmed influenza (74% had proven pandemic [H1N1] 2009) in Children’s Hospital at Westmead (CHW), Sydney, as part of a collaboration between the National Centre for Immunisation Research and Surveillance and the Australian Paediatric Surveillance Unit. The study was approved by the Human Research Ethics Committee at CHW and supported by the state (New South Wales) health department. Data from hospitalizations for seasonal influenza at CHW in 2003 and 2007 (previous peaks in the last decade) were analyzed by using our previous studies and medical records (*2**–**4*). To compare pneumonia rates, we used the same case definitions in 2007 and 2009 (radiologic changes consistent with pneumonia). Proportions were compared by using the χ^2^ statistic.

In 2009, the numbers of children with laboratory-confirmed influenza admitted to the hospital and to the pediatric intensive care unit (PICU) at CHW (226 and 22, respectively) were nearly double those admitted in 2007 (122 and 12) but similar to the number in 2003 (257 and 22). The proportion of case-patients admitted to the PICU, the length of hospital stay, and the length of PICU stay were similar in 2003, 2007, and 2009.

In 2009, among the 226 influenza-associated hospitalizations, 167 (74%) were for pandemic (H1N1) 2009 infection; in 2007, 119 of 122 influenza-associated hospitalizations were for seasonal influenza A (H3N2) infection (Table). During the 2009 pandemic wave, of all children admitted with laboratory-confirmed influenza, the proportion hospitalized with pandemic (H1N1) 2009 who were <6 months of age was similar to the proportion of children <6 months of age hospitalized with seasonal (H3N2) influenza in 2007 (21 [13%] of 167 and 21 [18%] of 119, respectively; p = 0.31). The proportions of those >5 years of age were significantly higher (61 [37%] and 15 [13%]; p = 0.0001). However, the proportion of those >5 years of age admitted to PICU in 2009 was less than in 2007 (10 [16%] of 61 vs. 3 [20%] of 15; p = 0.71). Similar percentages of children with preexisting conditions were admitted in 2009 and 2007 (47% and 49%, respectively). However, pneumonia was a more frequent complication in 2009 than in 2007 (42 [25%] of 167 vs. 15 [13%] of 119; p = 0.01). In 2009, the proportion of children with pandemic (H1N1) 2009 who needed mechanical ventilation (7 [4%] of 167) was similar to the proportion in 2007 who had seasonal influenza (H3N2) (6 [5%] of 119; p = 0.77). Furthermore, no child at CHW in 2007 or in 2009 received extracorporeal membrane oxygenation.

**Table:** Comparison of influenza-related hospitalizations, Children’s Hospital at Westmead, Sydney, Australia, 2003, 2007, and 2009 influenza seasons*

Characteristics†	2009, pandemic (H1N1) 2009, no. (%), n = 167	Seasonal influenza A (H3N2), no. (%)	
		2007, n = 119	2003, n = 257‡
PICU admissions	18 (10.8)	12 (10.1)	22 (8.6)
Fatal cases (within 30 d of hospital admission)	0	0	3 (1.2)
Ventilated	7 (4.2)	6 (5.0)	14 (5.4)
Treated with antiviral drug§	92 (55.1)	16 (13.4)	0
Symptoms			
Vomiting‡	59 (35.3)	16 (13.4)	NA
Diarrhea	21 (12.6)	8 (6.7)	NA
Seizure	11 (6.6)	7 (5.9)	NA
Complications			
Any complication	65 (38.9)	35 (29.4)	NA
Pneumonia¶	42 (25.1)	15 (12.6)	NA
Encephalopathy	5 (3.0)	2 (1.7)	NA
Preexisting condition	78 (46.7)	60 (50.4)	NA

*PICU, pediatric intensive care unit; NA, not available. †Length of hospital stay, mean (range), d: pandemic (H1N1) 2009, 5.9 (1–107); seasonal influenza in 2007, 4.1 (1–50); seasonal influenza in 2003, 4 (1–28). Length of PICU stay, mean (range), d: pandemic (H1N1) 2009, 3.7 (1–30); seasonal influenza in 2007, 4.3 (1–25); seasonal influenza in 2003, 3.3 (0.6–11.4) Data for length of PICU stay in 2003 exclude 3 deaths; 1.3, 2.3, and 4.8 d after PICU admission (2.8 d mean PICU stay). ‡Only isolates from the 22 PICU case-patients were subtyped; all were H3N2. §p = 0.0001. ¶p = 0.0104.

Vomiting occurred much more frequently in 2009 than in 2007 (59 [35%] of 167 vs. 16 [13%] of 119; p = 0.0001). In 2009, of 62 children who did not exhibit vomiting when first examined and who were subsequently treated with antiviral drugs, only 1 had vomiting develop in the hospital. This condition resolved within hours, and the 5-day course of antiviral treatment was completed. Chart review showed that in no child did antiviral treatment exacerbate vomiting, and no children required antiemetic treatment or intravenous rehydration. These data suggest that oseltamivir was uncommonly associated with vomiting in hospitalized children.

In macaque monkeys, pandemic (H1N1) 2009 virus is more pathogenic than seasonal influenza A (H1N1), particularly affecting the lungs (*5*). The significantly higher proportion (and number) of pneumonia patients at CHW in 2009 than in 2007 (Table) seems to provide additional evidence of the pathogenecity of pandemic (H1N1) 2009 virus. However, the number of children with laboratory-confirmed influenza admitted to the hospital was similar in 2009 and 2003. Given that the sensitivity of influenza laboratory tests has improved over time (e.g., greater use of nucleic acid tests), these data suggest that the incidence of hospitalization during the 2009 pandemic was not greater than the incidence during the 2003 influenza season.

Although pneumonia appeared more likely to be diagnosed in hospitalized children in 2009 than in 2007, we observed no increase in the risk for serious outcomes (PICU admission or ventilation rate, length of admission, or death) in children hospitalized with pandemic (H1N1) 2009 infection than in children hospitalized with seasonal influenza (H3N2) during the 2 peak years studied. The 2009 pandemic had an effect on children’s services that was comparable to the busiest interpandemic influenza season of the previous decade. The large number of admissions and complications, including in children with no existing medical condition, supports the need for universal influenza vaccination.
